# Effects of non-pharmacological coping strategies for reducing labor pain: A systematic review and network meta-analysis

**DOI:** 10.1371/journal.pone.0261493

**Published:** 2022-01-21

**Authors:** Ching-Yi Chang, Meei-Ling Gau, Chi-Jung Huang, Hao-min Cheng

**Affiliations:** 1 School of Nursing, College of Nursing, Taipei Medical University, Taipei, Taiwan; 2 Department of Nursing, Shuang Ho Hospital, Taipei Medical University, New Taipei City, Taiwan; 3 Department of Midwifery and Women Health Care, National Taipei University of Nursing and Health Sciences; 4 Center for Evidence-based Medicine, Taipei Veterans General Hospital, Taipei, Taiwan; 5 Program of Interdisciplinary Medicine (PIM), National Yang Ming Chiao Tung University College of Medicine, Taipei, Taiwan; 6 Institute of Public Health and Community Medicine Research Center, National Yang Ming Chiao Tung University College of Medicine, Taipei, Taiwan; 7 Division of Cardiology, Department of Medicine, Taipei Veterans General Hospital, Taipei, Taiwan; Illawarra Shoalhaven Local Health District, AUSTRALIA

## Abstract

**Background:**

Facilitating the childbirth process is a global issue. Many strategies have been developed to cope with labor pain and improve the delivery experience and satisfaction of pregnant women. The results of different types of medical intervention on women’s expectant pain have been varied. Therefore, this systematic review was aimed at summarizing the body of evidence regarding the effects of various non-pharmacological coping strategies for reducing labor pain.

**Methods:**

The review was conducted according to guidelines of the Preferred Reporting Items for Systematic Reviews and Meta-Analyses (PRISMA). We systematically searched the articles published between 1989 and 2020 in six electronic databases: PubMed, MEDLINE, CINAHL, WOS, PsycARTICLES, and Airiti Library, and the reference lists of the Clinical Trial Registry. Twenty studies were identified, with eight eligible studies included in the Bayesian network meta-analysis.

**Results:**

Eight studies with 713 participants were included in the meta-analysis with nine different non-pharmacological strategies for reducing labor pain. The traditional meta-analysis demonstrated that the non-pharmacological coping strategies were effective in reducing labor pain. Of these interventional strategies, the ranking probabilities analysis of the network meta-analysis suggested that the Bonapace Method may be the most effective strategy in reducing labor pain, followed by acupressure.

**Conclusions:**

Non-pharmacological coping strategies can reduce labor pain while maintaining an effective and satisfactory delivery experience. This systematic review, by synthesizing the body of evidence, demonstrated that non-pharmacological coping strategies are effective in reducing labor pain. Furthermore, as demonstrated in the network meta-analysis, the Bonapace Method, modulating birth pain by involving the father, is the most effective non-pharmacological intervention for reducing labor pain.

## Introduction

Alleviating labor pain and improving the childbirth experience has been ongoing goals worldwide for the past decade [1–3]. Most women experience a great deal of labor pain while giving birth, and professional support is not always helpful. Women have reported receiving positive and negative support from birthing professionals [4]. Women often choose different coping strategies to reduce labor pain, including both pharmacological and non-pharmacological, or more natural, strategies [5]. The non-pharmacological coping strategies refer to methods other than medication that is designed to reduce labor pain [9–16]. For example, previous studies have considered women’s views and experiences and investigated the effects of various strategies to reduce labor pain, such as epidurals [6,7], opioids [8], breathing and relaxation techniques, massage techniques, laboring in water [9], hot compress [10], ice compress, essential oils [11], acupressure and music therapy [12], yoga [13,14], aromatherapy [15], and labor support [16].

Although empirical evidence has shown that different types of medical intervention can reduce a woman’s expectant pain, the results have been varied. Some researchers have reported that various non-pharmacological analgesic methods can help women feel in control and that natural remedies are less effective than expected [17]. Others have found that women do not like the unexpected complications associated with medication [18]. These findings highlight that women have different experiences of suffering from different physical pain. Furthermore, women need to be given information about the risks and benefits of all available techniques for relaxation and pain reduction [19]. Thus, reducing pain during labor is a widespread critical issue, and the techniques used to achieve this aim merit further investigation. To make informed decisions for pain relief during delivery, summarizing the totality of the evidence in a systematic approach is an important and imminent task.

However, despite the many previous studies on labor pain reduction, there has been no comprehensive analysis of the studied techniques, which is significant because the non-pharmacological reduction of labor pain can improve childbirth satisfaction by promoting cognitive, physical, and psychological support during delivery [20,21]. Thus, childbirth education should include a variety of approaches which have been adopted in the past two decades, including non-pharmacological methods. This article features a systematic meta-analysis of previous published studies with the aim of providing suggestions and resources to help medical staff better care for women in labor.

The meta-analysis method is a comprehensive analysis of previous research results that can avoid the influence of measurement errors caused by a single study [22]. Compared with the traditional pairwise meta-analysis, a network meta-analysis is a statistical technique that combines the results of several studies (usually randomized trials) to compare several treatments or interventions [23]. In addition, one of the advantages of Bayesian network meta-analyses is that it is easy to know whether each variable is conditionally independent or dependent and its local distribution type, so as to obtain the joint distribution of all random variables. The comprehensive meta-analysis of labor pain techniques can obtain more general and accurate conclusions and is expected to provide strong evidence to inform clinicians as an evidence-based practice. Moreover, for the many interventional strategies in reducing birth pain, we conducted Bayesian network meta-analyses to determine the most effective pain-lowering method. This study raised the following research questions: (1) What are the conditions for designing and applying research to reduce labor pain? (2) What is the overall effect of each condition? (3) Are the study methods effective in reducing labor pain? (4) Which method is the most effective for treating birth pain?

## Methods

The methodology for this meta-analysis followed the Preferred Reporting Items for Meta-Analysis (PRISMA) guidelines [24,25]. The article search, review process, and analysis were documented in advance. We included randomized controlled trials (RCTs) in this review. We considered studies in which pain reduction methods were used at the labor stage of childbirth for inclusion in the review.

### Inclusion/Exclusion criteria

For inclusion, the studies were required to meet the following criteria:

1. non-pharmacological coping strategies were provided to explore the effect of reducing labor pain;
2. the research design included intervention and control groups;
3. the outcome measurement tool was the VAS and McGill Pain Questionnaire (SF-MPQ) Survey;
4. the studies had at least one relationship or other indicators that could be transformed into an effect size;
5. the articles were sample independent;
6. re-published articles were only chosen once and had to have been published in an academic journal;
7. the research subjects were women in labor for delivery;
8. the study data had a clear average and standard deviation; and
9. the articles were peer-reviewed publications.

The exclusion criteria were as follows:

1. the study did not adopt an experimental design;
2. the publication was not in English;
3. the studies were case studies, qualitative research, pure theory, or literature review articles; and
4. the studies were conference papers or book chapters and/or the full text was not accessible.

Although the exclusions may increase the risk of publication bias, we used standardized and widely accepted methods of calculating fail-safe numbers to avoid other unpredictable deviations [26].

### Study search process

This study comprehensively searched the science citation index (SCI) and social science citation index (SSCI) literature, and the articles included in the meta-analysis were screened according to the above inclusion/exclusion criteria. Studies were identified by searching the PubMed, Web of Science (WOS), CINAHL, Cochrane, MEDLINE system, PsycARTICLES, Airiti Library, and Index of the Taiwan Periodical Literature System databases. The terms "labor" and "reduce pain" were used as keywords for articles published from January 1989 to December 2020. The research designs were limited to RCTs or controlled clinical trials (CCTs).

### Outcome measure

Our outcome measure was the Visual Analog Scale (VAS) to assess pain conditions. As the VAS had different scales in the included studies, the data were all normalized to the 0–10 range.

### Study selection process

**Fig 1:**
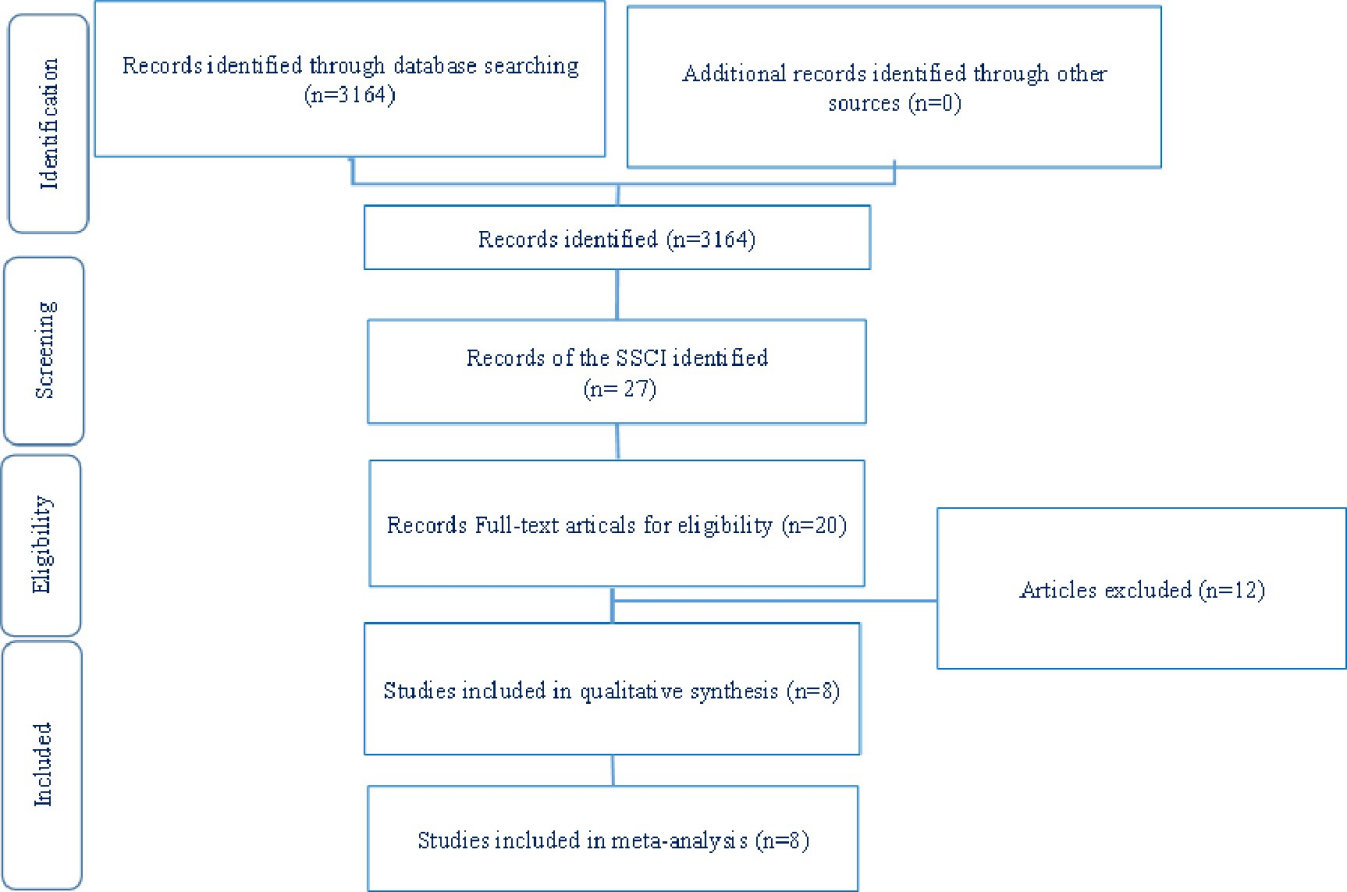
Flow diagram for study selection.

Two researchers (C.Y.C, M.L.G) searched the databases, identified duplicate articles, and excluded articles in which the participants were not women in labor for delivery. These two researchers screened the full-text articles to independently confirm the included articles, and the third researcher (C.J.H) made the final decision in the case of disagreement between the other two. Fig 1 shows the preferred reporting items for systematic reviews and meta-analyses (PRISMA) for searching and identifying the included studies. The results from the two independent reviewers regarding the suitability of articles were compared. If the meaning of the study title was unclear, the article was investigated further. Among the studies, 20 were related to labor pain articles. After removing 12 non-RCT or non-CCT papers, eight were selected in the final list for analysis, as shown in Fig 1.

Two authors (C.Y.C, M.L.G) independently reviewed the search output. We screened the titles and abstracts of search results to exclude irrelevant studies. We then retrieved the full-text articles of seemingly relevant studies and examined them to see whether they met the inclusion criteria. We resolved any disagreement through discussion and consensus.

### Data extraction and quality assessment

We designed a data extraction form to collect relevant information, including author, year of publication, country, participants, intervention, comparison, and outcome. We used the Cochrane risk of bias tool to judge the quality of the included studies. Publication bias was detected via a funnel plot visualization. The funnel plots were used to assess symmetry: if the sample sizes were small, the article would appear on the lower side; if the sample sizes were large, the article would appear on the higher side. An symmetrical plot indicates no publication bias. However, the small number of articles chosen for this study could affect the precision of the results [3]. Two review authors (C.Y.C, M.L.G) independently assessed the quality of the trial, extracted data, and checked the accuracy of the data. Disagreements were resolved by consensus.

### Statistical analysis

We conducted network meta-analysis for mixed treatment comparisons in a Bayesian framework and obtained the pooled estimates using the Markov chain Monte Carlo (MCMC) method, which is recommended by the National Institute for Health and Care Excellence (NICE) Decision Support Unit’s technical support documents on evidence synthesis. A random-effects network meta-analysis was performed with ADDIS Version 1.8 and GeMTC-GUI-0.14.3, which uses Bayesian MCMC methods with 50,000 times random sampling [27,28]. There are three parts to these analyses. First, in the network meta-analysis for the consistency model, all relative effects were estimated simultaneously by using the consistency constraint. For example, the parameter dBC was estimated from both direct evidence on BC and indirect evidence on AC and AB. We reported the relative effect results for the consistency model as an odds ratio (OR) with a corresponding 95% confidence interval (CI). Subsequently, the ranking probability was estimated for each technique, i.e., the most effective, the second -best, and so on. Using the surface under the cumulative ranking (SUCRA) technique ensuring the sum of rank probabilities equaled one, the overall ranks were interpreted. The most preferred agent for pain control is ranked as one treatment [29].

Second, the inconsistency analysis was performed using the inconsistency model and the node-splitting model to check whether the analysis of the trials in the network was indeed consistent. Inconsistency factors, representing the discrepancy between the direct and indirect evidence, were added to the closed loops of the inconsistency model, i.e., dBC = dAC − dAB + φ (where φ = inconsistency factor). Therefore, the degree of inconsistency was determined for a cycle (e.g., ABC) by checking the size of an inconsistency factor within the cycle rather than for individual pairwise comparisons. Previous studies suggested that when the 95% CI of the median of the inconsistency factors includes zero and if the inconsistency standard deviation is less than or equal to the random effect standard deviation, the inconsistency can be considered insignificant [29].

VAS was used as the outcome measure for the network meta-analysis. As significant heterogeneity was noted across studies, we also conducted a traditional pairwise meta-analysis using the random-effects model of DerSimonian and Laird. We quantified the I^2^ statistic to estimate the proportions of inconsistencies across the studies not explained by chance and performed Cochran’s Q test to evaluate the heterogeneity between subgroups. The traditional pairwise meta-analysis was conducted using CMA (version 2.2.064) and RevMan 4.

This study is a systematic review without any investigations that involve human subjects. There is no intervention or interaction with humans or collection of identifying private information. Therefore, the approval of the ethical committee for the study conduct has been waived.

## Results

### Study selection and study characteristics

Of the 20 identified articles, eight (encompassing 713 women) contributed suitable data for the meta‐analysis. Twelve articles were excluded because they did not adopt an experimental design, were not published in English, lacked qualitative research, were pure theory, were literature review articles, or did not have the full text. Table 1 summarizes the characteristics of the included trials. The eight studies were published between 2006 and 2019. The studies were from the United Kingdom [30], Sweden [31], Taiwan [32], Iran [33,34], Canada [35], France [36], and Turkey [37]. All the studies evaluated participants immediately after the interventions. There were six RCTs [30–35] and two quasi-experimental studies [36,37].

**Table 1:** Characteristics of studies (n = 8).

Author(s), year	Participants	Country	Coping strategies	Design	No. of subjects	EE mean (SD)	CP mean (SD)
Sanders, Campbell, and Peters (2006) [30]	Pregnant women	United Kingdom	Lidocaine spray	RCT	E = 91 C = 90	76.9** (21.6)	72.1** (22.2)
Hjelmstedt et al. (2010) [31]	Pregnant women	Sweden	Acupressure	RCT	E = 71 C = 71	74** (18.2)	78.9** (19.9)
Gau (2011) [32]	Pregnant women	Taiwan	Birth ball exercise	RCT	E = 48 C = 39	3.2 (0.7)	4 (0.7)
Hamidzadeh et al. (2012)	Pregnant women	Iran	LI4 acupressure	RCT	E = 50 C = 50	6.3* (1.39)	8.3* (1.4)
Bonapace et al. (2013)	Pregnant women	Canada	Bonapace Method: a specific educational intervention	RCT	E = 13 C = 12	44.14 (16.08)	79.73 (25.81)
Guétin et al. (2018)	Pregnant women	France	Smartphone-based music intervention	CCT	E = 19 C = 19	3.68* (2.99)	2.95* (3.22)
Yildirim, Alan, and Gokyildiz (2018)	Pregnant women	Turkey	Ice pressure	CCT	E = 36 C = 36	8.61* (NR)	5.25* (NR)
Amiri et al. (2019)	Pregnant women	Iran	Distraction techniques	RCT	E = 34 C = 34	6.2* (1.4)	7.5* (1.4)

E: Experimental group; C: Control group; EE: Experimental group’s mean and SD; CP: Control group’s mean and SD

*Visual analogue scale with range 0–10

**Visual analogue scale with range 0–100.

### Risk of bias in included studies

**Fig 2:**
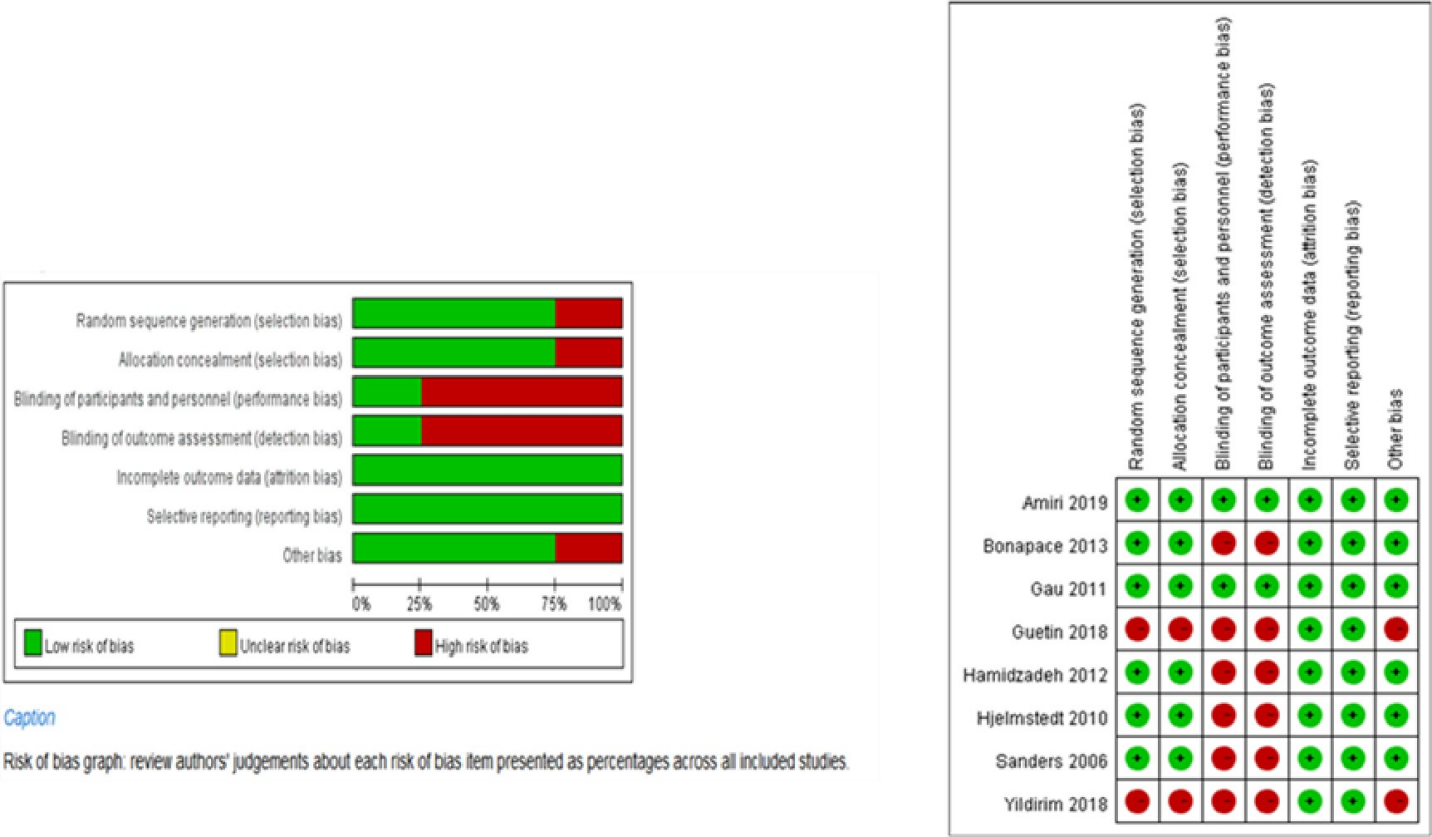
Risk of bias graph for studies.

**Fig 3:**
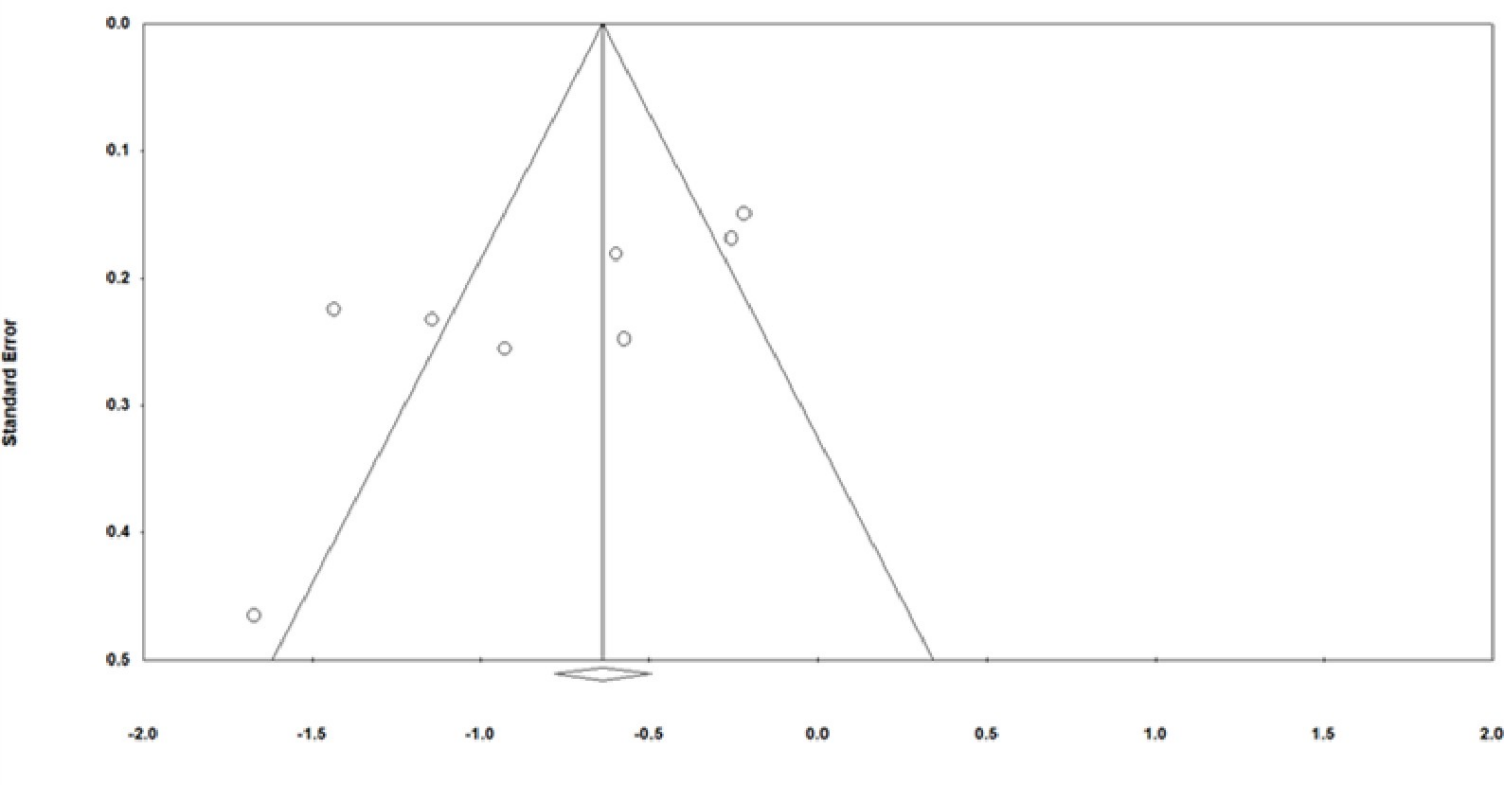
Funnel plot of included studies on reducing labor pain.

Fig 2 presents the evaluation results for the quality of the studies. Six of the eight studies included in this meta-analysis have a high risk of bias due to non-blinding of participants and personnel (performance bias) and the non-blinding of outcome assessment (detection bias). Fig 3 shows the funnel plots for the standardized mean differences (SMD) of reduced labor pain and log odds ratios. As the graph is symmetrical on both sides, there was no evidence of publication bias. Most of the studies had minor standard errors, indicating high study accuracy.

### Results of individual studies and synthesis of results

**Fig 4:**
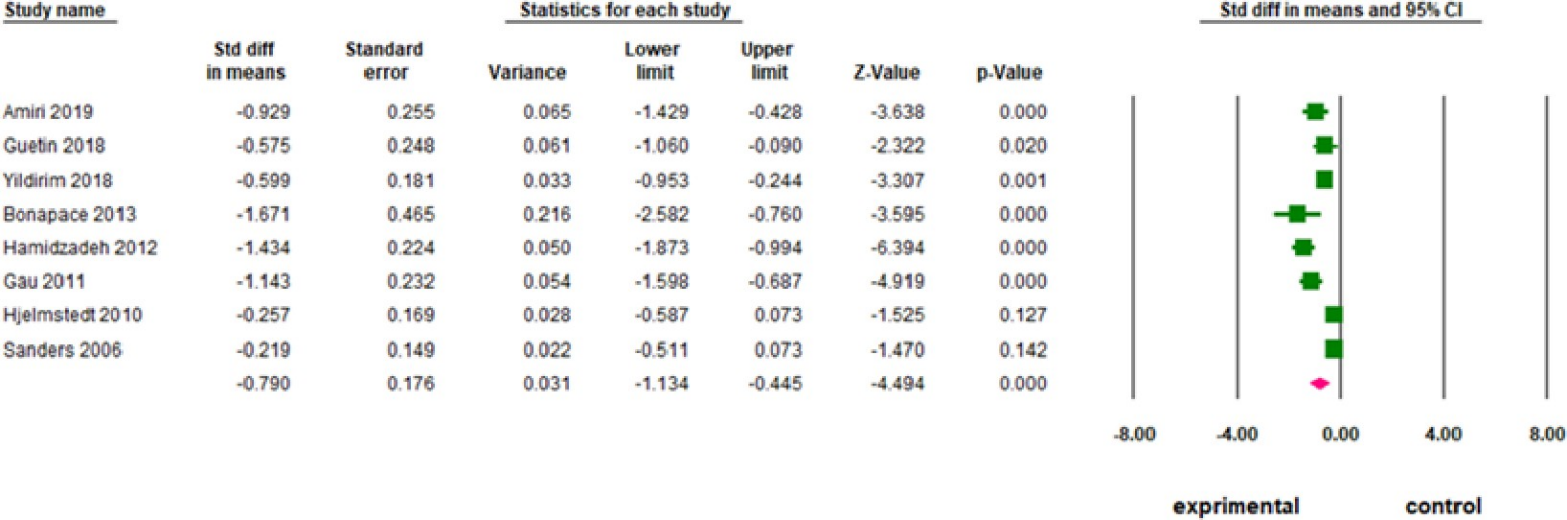
The effect of non-pharmacological interventions for reducing labor pain.

Across the eight studies, there were 713 participants (362 in experimental groups and 351 in control groups) included in the meta-analysis. The publication years ranged from 2006 to 2019. The sample sizes ranged from 25 to 181 participants (Table 1). Because only eight studies were included in the analysis, it was difficult to examine the risk of bias. The results of the included studies showed that there was no heterogeneity among the samples. Fig 4 displays the pooled estimates of effect comparisons between the nonpharmacologic interventions and standard pain-lowering treatments. Compared with the standard regimens, nonpharmacologic interventions for pain relief showed a significant reduction in labor pain (mean difference, -0.79; 95% CI, -1.13 to -0.45 cm, *p* < .0001). In summary, six studies showed a significant difference in reducing labor pain. However, two studies [30,31] found non-pharmacological strategies had no statistically significant different pain-lowering effect (*p* = .14; 95% CI: -0.51, 0.07 and *p* = 0.13; 95% CI: -0.59, 0.07, respectively). The methods used by these studies to reduce labor pain, including lidocaine spray [30] and acupressure [31] may not be the best interventions.

### Results of bayesian network meta-analysis

**Fig 5:**
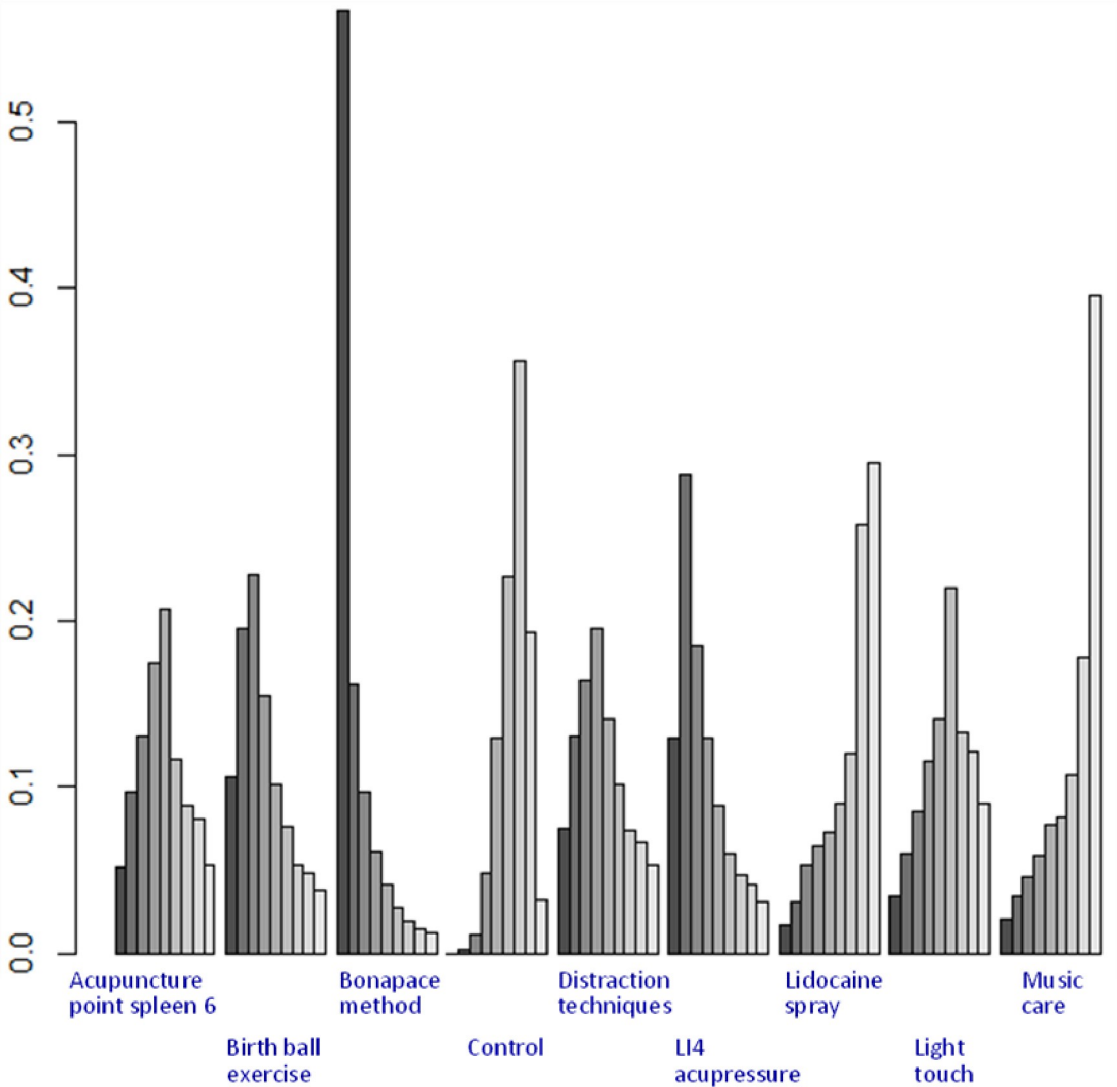
Ranking of coping strategies based on the probability of their effects on the reduction of labor pain. The horizontal axis represents the possible ranks of preferred regimens (from left to right) for each technique. The vertical axis shows the ranking probabilities.

The results in Table 2 show that no statistical significance for the mean difference between regimens was observed for all pairwise comparisons. However, pain-lowering using the Bonapace Method appeared to have the lowest VAS scores among the studied techniques. In the probability ranking analysis (Table 3 and Fig 5), the Bonapace Method showed a higher probability of being in the best-ranking positions (95.4%), followed by LI4 acupressure (3.7). Music care was the coping strategy with the highest probability (59.3%) of being in the last ranking position.

**Table 2:** Network estimated mean differences (95% CI) in VAS scores of labor pain between regimens.

Acupuncture point spleen 6	-0.6683 (-1.423, 0.0880)	-2.517 (-4.34, -0.7415)	1.033 (0.4669, 1.59)	-0.2699 (-1.139, 0.5947)	-0.9664 (-1.753, -0.1966)	1.516 (0.6665, 2.353)	0.4878 (-0.1481, 1.124)	1.772 (-0.3469, 3.807)
0.6683 (-0.0880, 1.423)	Birth ball exercise	-1.857 (-3.643, -0.0705)	1.702 (1.199, 2.212)	0.4013 (-0.4332, 1.22)	-0.2955 (-1.045, 0.455)	2.183 (1.36, 2.998)	1.158 (0.3739, 1.942)	2.436 (0.3335, 4.443)
2.517 (0.7415, 4.34)	1.857 (0.0705, 3.643)	Bonapace method	3.556 (1.865, 5.266)	2.252 (0.4286, 4.1)	1.551 (-0.2102, 3.345)	4.034 (2.224, 5.863)	3.007 (1.215, 4.83)	4.296 (1.647, 6.874)
-1.033 (-1.59, -0.4669)	-1.702 (-2.212, -1.199)	-3.556 (-5.266, -1.865)	Control	-1.301 (-1.967, -0.6432)	-2.0001 (-2.542, -1.457)	0.4798 (-0.1622, 1.118)	-0.545 (-1.129, 0.0527)	0.7386 (-1.306, 2.68)
0.2699 (-0.5947, 1.139)	-0.4013 (-1.22, 0.4332)	-2.252 (-4.1, -0.4286)	1.301 (0.6432, 1.967)	Distraction techniques	-0.7012 (-1.558, 0.159)	1.778 (0.8669, 2.701)	0.7546 (-0.1267, 1.646)	2.035 (-0.0830, 4.104)
0.9664 (0.1966, 1.753)	0.2955 (-0.455, 1.045)	-1.551 (-3.345, 0.2102)	2.0001 (1.457, 2.542)	0.7012 (-0.159, 1.558)	LI4 acupressure	2.48 (1.635, 3.324)	1.459 (0.6566, 2.262)	2.727 (0.6115, 4.785)
-1.516 (-2.353, -0.6665)	-2.183 (-2.998, -1.36)	-4.034 (-5.863, -2.224)	-0.4798 (-1.118, 0.1622)	-1.778 (-2.701, -0.8669)	-2.48 (-3.324, -1.635)	Lidocaine spray	-1.023 (-1.891, -0.1536)	0.2576 (-1.861, 2.294)
-0.4878 (-1.124, 0.1481)	-1.158 (-1.942, -0.3739)	-3.007 (-4.83, -1.215)	0.545 (-0.0527, 1.129)	-0.7546 (-1.646, 0.1267)	-1.459 (-2.262, -0.6566)	1.023 (0.1536, 1.891)	Light touch	1.288 (-0.8327, 3.315)
-1.772 (-3.807, 0.3469)	-2.436 (-4.443, -0.3335)	-4.296 (-6.874, -1.647)	-0.7386 (-2.68, 1.306)	-2.035 (-4.104, 0.0830)	-2.727 (-4.785, -0.6115)	-0.2576 (-2.294, 1.861)	-1.288 (-3.315, 0.8327)	Music care

**Table 3:** Ranking probabilities for the effectiveness of different coping strategies in pain relief during labor.

Intervention	[,1]	[,2]	[,3]	[,4]	[,5]	[,6]	[,7]	[,8]	[,9]
Acupuncture point spleen 6	0.0000	0.0031	0.0287	0.2409	0.6321	0.0903	0.0048	0.0001	0.0002
Birth ball exercise	0.0075	0.2085	0.5938	0.1659	0.0226	0.0015	0.0002	0.0002	0.0000
Bonapace method	**0.9544**	0.0258	0.0126	0.0052	0.0017	0.0004	0.0001	0.0000	0.0000
Control	0.0000	0.0000	0.0000	0.0001	0.0002	0.0251	0.6934	0.2661	0.0152
Distraction techniques	0.0011	0.0372	0.1537	0.5271	0.2307	0.0473	0.0031	0.0001	0.0000
LI4 acupressure	0.0367	0.7209	0.2047	0.0354	0.0024	0.0001	0.0000	0.0000	0.0000
Lidocaine spray	0.0000	0.0000	0.0000	0.0000	0.0003	0.0058	0.0546	0.5489	0.3905
Light touch	0.0000	0.0000	0.0008	0.0110	0.0812	0.7654	0.1278	0.0125	0.0015
Music care	0.0004	0.0047	0.0060	0.0146	0.0290	0.0642	0.1162	0.1723	**0.5927**

### Comparisons between traditional pairwise and Bayesian network meta-analyses

The inconsistency analysis using the inconsistency model showed no significant inconsistency within the networks.

### Discussion

Our findings suggest that the non-pharmacological coping strategies, as shown in the traditional meta-analysis, effectively reduced labor pain. Furthermore, as demonstrated in the network meta-analysis, the Bonapace Method is the most effective non-pharmacological intervention for reducing labor pain. Although none of the between-intervention comparisons demonstrated significant differences, the probability ranking analysis suggested that Bonapace Method-based interventions should be the preferred non-pharmacological treatment to prevent labor pain (Table 2).

The results of this meta-analysis can support patients and healthcare professionals in choosing the most effective techniques to reduce labor pain. Because patients with labor pain tend to have medical treatment, it is important to identify effective interventional strategies to reduce labor pain based on personalized suggestions for lower-risk delivery [38]. Our study is the most comprehensive systematic review and meta-analysis of labor pain reduction that combines direct and indirect comparison through the construction of complex networks (Table 3). Via the network meta-analysis, we were able to make indirect comparisons between interventions and determined the relative differences between different intervention strategies.

Our study demonstrated that the Bonapace Method is the most preferred strategy to reduce labor pain. The Bonapace Method involves the father or a significant partner in helping to reduce labor pain by practicing pain modulation techniques based on neurophysiological endogenous pain modulation models. Our study findings were also in agreement with previous reviews [39,40,41], which demonstrated that non-pharmacological coping strategies are effective in relieving labor pain. The decrease in labor pain is related to the pain threshold [6,35]. Thus, we consider that the main effects of these coping strategies are to increase the women’s pain thresholds in labor.

### Impact

Many women would prefer to avoid pharmacological or invasive methods to manage labor pain, which may contribute to the popularity of alternative strategies. This systematic review examined the evidence currently available on non-pharmacological methods, including massage and reflexology, for labor pain management.

The purpose of the present study was to examine the effects of coping strategies on diminishing and maintaining reduced labor pain for women using a meta-analysis of previous studies. We found that coping strategies can decrease labor-related pain. The coping strategies that were found to be effective included birth ball exercise, LI4 acupressure, the Bonapace Method, smartphone-based music, ice pressure, and distraction techniques. During labor, uterine contraction effectively reduces pain, while personal confidence is only effective during delivery [42]. Although strategies for decreasing labor pain include giving pregnant women more emotional control, the continued cognitive, physical, and psychological support provided to women during delivery may be the most important coping strategy for reducing labor pain [20,21].

### Limitations and strengths

Our meta-analysis has some limitations. First, similar to other meta-analyses, the absence of primary data and the selective reporting of primary studies may confound our study results. Second, despite the comprehensive literature search, we may have failed to locate eligible published or unpublished studies. However, like trends reported in previous meta-analyses [43], the study conclusions would likely not be altered substantially if there were some un-retrieved studies. The number of studies analyzed was limited (n = 8), which may lead to insufficient evidence. Although the study design focused on RCTs or CCTs, most researchers did not describe methods for assessing adverse reactions, leading to information gaps on adverse effects of the studied techniques. In addition, it was difficult to adopt a blinding method. More studies with different coping strategies should be explored in the future.

Further, the scope of this review, such as only including English-language papers, may have limited the available studies. The keywords may be not broad enough, which may have led to some relevant articles being missed. We recommend that future research include broader keywords and empirical articles for further analysis and discussion.

## Conclusions

Labor pain is a critical issue that may be reduced or eliminated with an effective coping strategy, varying for different women. This meta-analysis provides evidence that non-pharmacological coping strategies can reduce labor pain and should be included in early intervention. The non-pharmacological management of pain during labor can improve childbirth satisfaction^.^[44]. Thus, interventions to reduce pain should be implemented at regular intervals during labor. Further, interventions should include prenatal education of alternative strategies to provide effective coping strategies.

Evidence from randomized controlled trials supports the use of the Bonapace Method for reducing labor pain. The Bonapace Method, especially when combined with the standard pain-lowering treatments, supports the secondary preclusion of labor pain. With this comprehensive meta-analysis, clinicians and women can make evidence-based decisions regarding chosen techniques to reduce labor pain.

## Supporting information

S1 Checklist PRISMA checklist. (DOC)
